# Protamine-stabilized RNA as an ex vivo stimulant of primary human dendritic cell subsets

**DOI:** 10.1007/s00262-015-1746-9

**Published:** 2015-08-15

**Authors:** Annette E. Sköld, Jasper J. P. van Beek, Simone P. Sittig, Ghaith Bakdash, Jurjen Tel, Gerty Schreibelt, I. Jolanda M. de Vries

**Affiliations:** 1 Department of Tumor Immunology, Radboud Institute for Molecular Life Sciences, Radboud University Medical Centre, Nijmegen, The Netherlands; 2 Department of Oncology-Pathology, Karolinska University Hospital Solna, Karolinska Institutet, Stockholm, Sweden; 3 Department of Medical Oncology, Radboud University Medical Centre, Geert Grooteplein 26, Route 278, 6525 GA Nijmegen, The Netherlands

**Keywords:** Immunotherapy, pDC, mDC, TLR, Adjuvants, GMP

## Abstract

Dendritic cells (DCs) are key in connecting innate and adaptive immunity. Their potential in inducing specific immune responses has made them interesting targets for immunotherapeutic approaches. Our research group was the first to exploit the naturally occurring myeloid DCs (mDCs) and plasmacytoid DCs (pDCs) in therapeutic vaccination trials against melanoma. To develop primary DC subsets as an optimal vaccine, the identification of a clinically applicable adjuvant activating both subsets is required. Although the expression of pathogen recognition receptors differs distinctly between the DC subsets, both pDCs and mDCs can respond to single-stranded RNA (ssRNA) via Toll-like receptors 7 and 8, respectively. Since ssRNA is easily degraded by RNases, we stabilized anionic RNA by complexing it with the positively charged protein protamine. This leads to the formation of protamine–RNA complexes with varying features depending on ionic content. We subsequently investigated the immunostimulatory effect of complexes that formed various salt concentrations on purified DC subsets. Both mDCs and pDCs upregulated maturation markers and produced pro-inflammatory cytokines in a dose-dependent way to the protamine–RNA complexes. This was dependent on endosomal acidification and correlated partly with the uptake of protamine–RNA complexes. Furthermore, both DC subsets induced T cell proliferation and IFN gamma secretion in a beneficial ratio to IL-10. These results indicate that protamine–RNA complexes can be used to stimulate human mDC and pDC ex vivo for use in immunotherapeutic settings.

**Electronic supplementary material:**

The online version of this article (doi:10.1007/s00262-015-1746-9) contains supplementary material, which is available to authorized users.

## Introduction

Dendritic cells (DCs) connect the innate and the adaptive immune systems. In blood, three major DC subsets are found: CD1c^+^ (BDCA1^+^) myeloid DCs (mDCs), CD141^+^ (BDCA3^+^) mDCs, and CD303 (BDCA2^+^) plasmacytoid DCs (pDCs) [1, 2]. After a DC has encountered an antigen, it matures and migrates to the adjacent lymph node, where it interacts with naïve T cells and initiates an antigen-specific immune response. The activation state of the DCs will influence the resulting adaptive response, spanning from tolerogenic to immunogenic [3]. To sense danger and to be able to initiate eradicating adaptive responses against antigens, DCs are equipped with a wide range of pattern recognition receptors (PRRs) [4]. These receptors recognize danger-associated molecular patterns, such as conserved microbial structures or nucleic acids, which upon binding mature the DCs. The types of PRRs being engaged on the DCs have a great impact on the subsequent adaptive immune response.

There are several groups of PRRs, the first characterized, and so far the most studied group, are the Toll-like receptors (TLRs) [5]. These are transmembrane molecules expressed in hetero- or homodimers, either on the cell surface or in the endosomal compartment. Most innate immune cells express TLRs in various combinations, and the receptor family therefore poses as potential target for vaccine adjuvants. In addition to the naturally occurring ligands, several synthetic TLR ligand analogs have been developed.

Due to their unique ability to stimulate naïve T cells, DCs are often used in cell-based immunotherapy. The majority of DC-based clinical studies are performed with in vitro differentiated DCs, such as monocyte-derived DCs or cells generated from CD34^+^ progenitor cells [6–8]. Our research group is using pDCs [9] and CD1c^+^ DCs (Schreibelt et al. manuscript in preparation) in therapeutic vaccination of melanoma patients, with promising results [9]. However, the stimuli used to mature these subsets are not purified PRR ligands, which are required for optimal activation of DCs [3]. Activation of PRRs such as TLRs will upregulate major histocompatibility complex (MHC) molecules, co-stimulatory molecules, and prime DCs to produce T helper (Th) polarizing cytokines. These cytokines act on interacting antigen-specific T cells and skew the adaptive immune response toward a desirable phenotype, depending on initial stimuli [10]. The usage of strong DC activators, such as TLR ligands, as adjuvant in immunotherapy is therefore desirable.

For effective anti-tumor effects, a Th1 response with the ability to activate cytotoxic CD8^+^ T cells (CTLs) is required. This is induced by interleukin (IL) 12p70-producing DCs, but can also be mediated by type I interferons (IFNs) [11, 12]. An inducer of IL-12p70 in mDCs is the TLR3 ligand poly I:C, while pDCs are secreting high levels of IFN-α in response to TLR9-activating CpG oligonucleotides [13]. However, CpG has been shown to inhibit the effect of poly I:C, and using a combination of these two ligands in an adjuvant would most likely not be beneficial [14]. Instead, to prevent unforeseen cross-reactions, a stimulus with the potency to activate both mDCs and pDCs is preferred. Additionally, a standardized protocol would also provide more flexibility in combining the two subsets in future studies and clinical trials. Cross-talk between mDCs and pDCs has been shown to be important both in anti-viral responses and during anti-cancer immunotherapy [15]. We hypothesize that using both subsets will result in a broader and multifaceted immune reaction in response to DC-based immunotherapy.

One obstacle for using one stimulus for both DC subsets is that they do not express an overlapping repertoire of TLRs [13]. However, TLR7 and TLR8, expressed by pDCs and mDCs, respectively, can respond to the same type of ligand—single-stranded RNA (ssRNA) [5, 16]. Unprotected ssRNA is a suboptimal ex vivo DC stimulator due to its sensitivity to RNases, RNA-degenerating enzymes present in, e.g., serum [17]. So far, there is no ligand targeting both TLR7 and TLR8 approved for clinical use other than topical application [18]. By using the polybasic protein protamine, ssRNA can be stabilized in an immunostimulatory protamine–RNA complex [19–21]. In this study, we have evaluated the effect of protamine–RNA complexes, consisting of clinically applicable reagents, on purified DC subsets. Both pDCs and CD1c^+^ DCs upregulated maturation markers and secreted pro-inflammatory cytokines upon treatment with protamine–RNA. This was dependent on endosomal maturation and the ability of the complexes to engage TLR signaling. Furthermore, protamine–RNA-stimulated DCs induced T cell proliferation and antigen-specific T cell activation, making the complexes a highly interesting stimulus for future vaccination trials based on primary human DC subsets.

## Materials and methods

### Reagents

As ligands for TLR3, 7/8, and 9, polyinosine-polycytidylic acid (poly I:C, 20 µg/ml; Sigma-Aldrich, St Louis, MO), imidazoquinoline (R848, 4 µg/ml; Axxora, San Diego, CA), and CpG class C DNA oligodeoxynucleotides (CpG, 5 µg/ml; Axxora) were used. To inhibit endosomal acidification, chloroquine (20 µM; Invivogen, Toulouse, France) was pre-incubated with the cells 1 h before addition of the stimulus. To capture cytokines intracellularly, brefeldin A (10 μg/ml; Sigma-Aldrich) was added to the cultured cells 12 h before analysis.

### Preparation of protamine–RNA complexes

To form the protamine–RNA complexes, protamine (protaminehydrochloride MPH 5000 IE/ml; Meda Pharma BV, Amstelveen, the Netherlands) was diluted to 0.5 mg/ml in water, 25 mM NaCl, or 50 mM NaCl and mixed 2:1 with ca 2-kbp-long single-stranded mRNA (coding for gp100, tyrosinase, or CEA, 0.5 mg/ml; CureVac GmbH, Tübingen, Germany). After extensive mixing, the mix was incubated for 5–10 min at room temperature and added to the cell cultures in the indicated concentrations. Dynamic light scattering and zeta potential of the complexes were measured in a Malvern Zetasizer 2000 (Malvern Instruments Ltd, Malvern, UK).

For the uptake experiments, the formed complexes were diluted 1:1 in the Fixable Viability Dye eFluor 780 (live–dead dye, 200X; eBioscience, San Diego, CA) and incubated at room temperature for 15 min. The reaction was stopped by addition of 10 % human serum or RNase-free bovine serum albumin and the labeled complexes were added to DCs. The DC viability was investigated by adding propidium iodide (500 ng/ml; Biolegend, San Diego, CA) just before acquisition.

### Cell isolation and culture

Peripheral blood mononuclear cells (PBMCs) were isolated from buffy coats of healthy individuals taken after informed consent using Ficoll density centrifugation (Lymphoprep; Axis-Shield PoC AS, Oslo, Norway). For CD1c^+^ DC and pDC isolation from PBMCs, microbead isolation kits were used (BDCA1^+^ DC and BDCA4^+^ DC isolation kits; Miltenyi Biotec, Bergisch-Gladbach, Germany), resulting in up to 95 % purity. During purification of CD1c^+^ DC, CD14^+^ cells were depleted using CD14 microbeads (Miltenyi Biotec). Prior pDC isolation, peripheral blood leukocytes (PBLs) were prepared by depleting monocytes from PBMCs either by plastic adherence or with CD14 microbeads. Isolated cells were cultured overnight at 5 × 10^5^ cells/ml in X-VIVO-15 medium (Cambrex, Verviers, Belgium) supplemented with 2 % human serum (Sanquin, Nijmegen, the Netherlands). For unstimulated pDCs, recombinant human IL-3 (10 ng/ml; Cellgenix, Freiburg, Germany) was added as a survival factor. T cells were isolated using negative microbead selection (Miltenyi Biotec), resulting in up to 98 % purity.

293XL-hTLR8 HEK cell lines (InvivoGen) expressing endosomal TLR8 were cultured in DMEM with GlutaMAX (Life Technologies, Carlsbad, CA) supplemented with 10 % fetal calf serum (FCS), 1 % antibiotic antimycotic (AA; PAA laboratories, Pasching, Austria), and blasticidin (10 µg/ml; InvivoGen) as selection antibiotic.

Jurkat E6.1 fl296 cells transfected with T cell receptor (TCR) v-beta14, as previously described [22, 23], were cultured in RPMI (Life Technologies) supplemented with 10 % FCS and 0.5 % AA.

### Stimulation with protamine–RNA complexes

All DC experiments were performed in X-VIVO-15 in the presence of 2 % human serum. For DC co-culture experiments, Jurkat cells were diluted in X-VIVO-15 supplemented with 2 % human serum. 293XL-hTLR8 HEK cell lines were stimulated in DMEM with GlutaMAX supplemented with 10 % FCS 1 % AA and blasticidin. protamine–RNA complexes were made fresh 5–10 min before addition to cell culture. R848 was used as TLR7/8 control.

### Flow cytometry

The purity of freshly isolated CD1c^+^ DC and pDC was assessed by staining with the following primary monoclonal antibodies (mAbs): anti-CD19-FITC (Dako, Glostrup, Denmark), anti-BDCA1-PE, anti-BDCA-2-APC (both Miltenyi Biotec), and anti-CD14-PerCP (BD Biosciences, San Jose, CA). The purity of freshly isolated T cells was determined by staining with mAb anti-CD20-FITC, anti-CD3-PE, and anti-CD56-APC (all BD Biosciences). For human leukocyte antigen (HLA) phenotyping, the mAb anti-HLA-A2-PE (BD Biosciences) was used. The samples were acquired in a FACSCalibur (BD Biosciences).

To stain for DC maturation, the following mAbs were used: anti-HLA-ABC-PE, anti-HLA-DR-FITC, anti-CD80-PE or PE-Cy7, anti-CD86-APC (all BD Biosciences), anti-HLA-DR-PerCP (Biolegend), and anti-CD40-PE (Immunotech, Marseille, France). To stain for C-C chemokine receptor type 7 (CCR7), anti-CCR7 mouse IgG2a (R&D Systems, Minneapolis, MN), and goat anti-mouse-IgG2a-Alexa647 or Alexa488 (Life Technologies) were used. Dead cells were detected with eFluor 780 live–dead cell marker (2000X). The samples were measured on a FACSCalibur or a CyAn ADP (Beckman Coulter, Fullerton, CA).

To assess cytokine production by T cells after stimulation with DCs, co-cultures were stained with eFluor 780 live–dead cell marker and the following mouse mAbs: anti-CD3-PE and anti-IFN-γ-APC (all BD Biosciences) or anti-IgG1-APC (eBioscience) as isotype control (data not shown). Prior to IFN-γ staining, cells were fixed and permeabilized using a cytofix/cytoperm kit (BD Biosciences). To exclude DC from the gated cell population, co-cultures were also stained with anti-BDCA2-PE-Cy7 (Biolegend) or APC (Miltenyi Biotec), and anti-CD11c-PE-Cy7. The activation of Jurkat cells was assessed with eFluor 780 live–dead dye, anti-CD3-FITC, and anti-CD69-APC (both BD Biosciences). The samples were measured on a CyAn ADP.

All analyses were performed using FlowJo Software (TreeStar Inc, Ashland, OR). Only viable cells gated on the specific population in forward-side scatter were assessed. The results are depicted either as percentage positive cells or as geometric mean fluorescence intensity (MFI) normalized to the negative control to compensate for use of different flow cytometers and fluorophores.

### Cytokine detection

Supernatants of stimulated cells were taken at indicated time points and analyzed with standard sandwich ELISAs detecting IL-12p70, IFN-γ (both from Thermo Scientific, Waltham, MA), IFN-α (Bender Medsystems, Vienna, Austria), IL-5, IL-10 (both from eBioscience), tumor necrosis factor (TNF) α, and IL-8 (both from BD Biosciences).

### Mixed lymphocyte reaction

The ability of stimulated DCs to induce T cell proliferation was investigated in a mixed lymphocyte reaction (MLR). Allogeneic PBLs or T cells were stained with carboxyfluorescein diacetate succinimidyl ester (CFSE, 5μM; Life Technologies) for 10 min; thereafter, the reaction was stopped by protein blocking using FCS. Overnight-activated DCs were cultured with CFSE-labeled cells in a 1:10 ratio for 3–5 days. As a control, Staphylococcal enterotoxin B (SEB, 5 μg/ml; Sigma-Aldrich) was used.

### Antigen-presentation assay

Donors were screened for HLA-A0201, and DCs from positive donors were stimulated with indicated stimuli, pulsed with 10 µM specific peptide (gp100_280–288_) or irrelevant peptide (tyrosinase_369-376_), and co-cultured 1:2 with Jurkat E6.1 fl296 cells expressing the TCR-v-beta14 overnight.

### Statistical analyses

To detect statistical significant differences between indicated groups, Wilcoxon matched-pair signed-rank tests or *t* tests were performed on raw data and paired measurements and analyzed with GraphPad Prism (GraphPad, La Jolla, CA). Values of *p* < 0.05 were considered significant.

## Results

### Protamine complexed with RNA forms positively charged particles with varying size depending on salt concentration

**Fig. 1:**
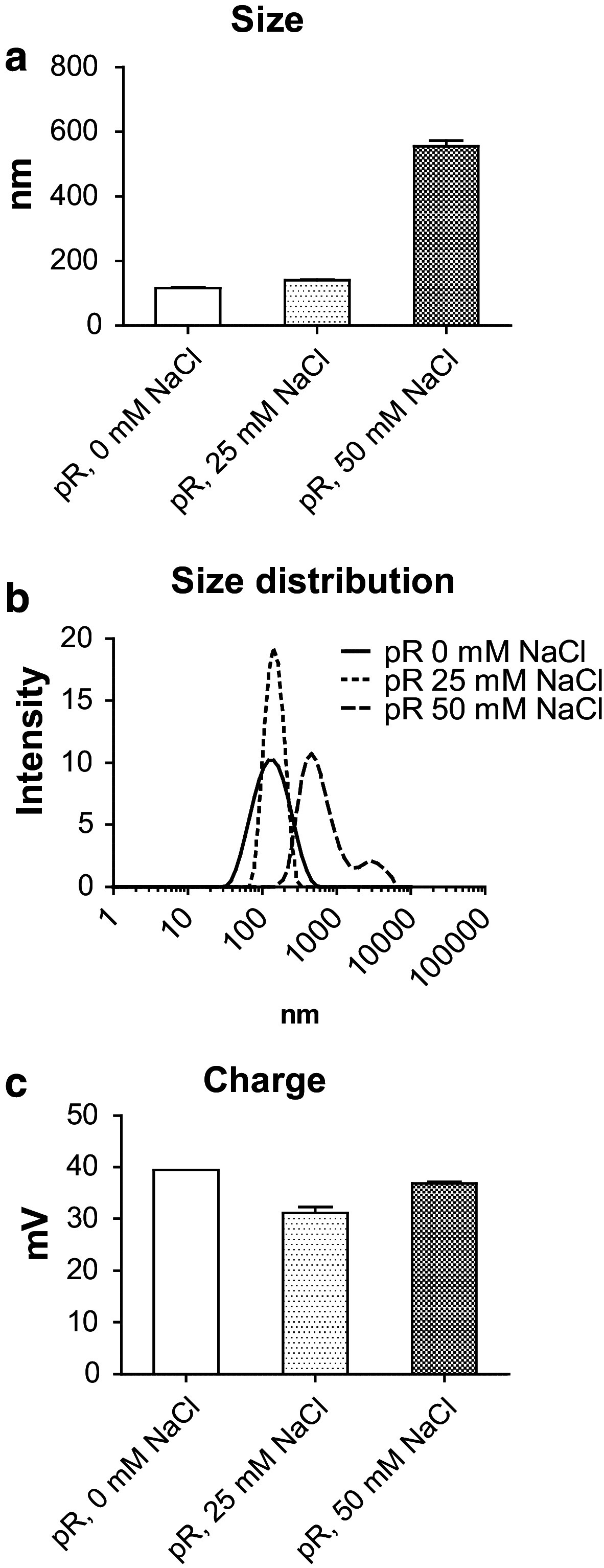
Concentration of NaCl determines size, but not charge, when forming protamine–RNA complexes. protamine–RNA complexes (pR) were formed in water, 25 mM NaCl, or 50 mM NaCl, particle size was evaluated by dynamic light scattering (**a**, **b**), and the zeta potential was investigated to determine the charge of the complexes (**c**)

It has long been known that negatively charged RNA has the ability to bind strongly to substances with positive charge [24]. A protein that has been used to complex nucleic acids is the polybasic protein protamine [17, 21, 25]. The physical characteristics of complexes formed when mixing anionic RNA to cationic protamine is dependent on the ratio between the components and on ionic content [26]. Since CD1c^+^ DCs and pDCs differ in their ability to take up and respond to particles [27] and express different TLRs [4], we produced protamine–RNA complexes in a ratio of 2:1 in 0, 25, or 50 mM NaCl and investigated their size by dynamic light scattering (Fig. 1a–b). The complexes formed in water or low salt concentration were <200 nm in diameter, while higher salt concentration formed complexes of >500 nm. The particle charge remained relatively constant between the formulations, ranging between 30 and 40 mV (Fig. 1c).

### Protamine–RNA complexes mature both CD1c^+^ DCs and pDCs in a concentration-dependent manner

**Fig. 2:**
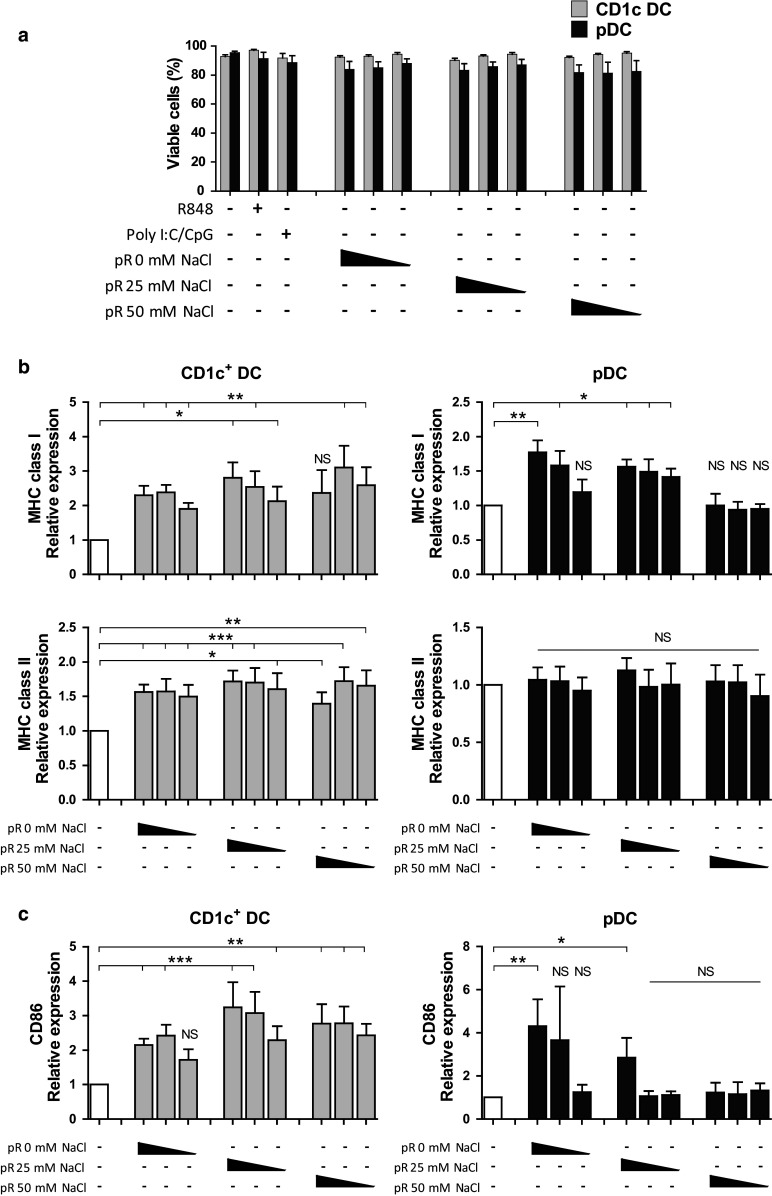
Protamine–RNA complexes are well tolerated by DCs and induce upregulation of maturation markers and MHC complexes. Purified CD1c^+^ DCs and pDCs were cultured 18–24 h with 15, 7.5, or 1.5 µg/ml of protamine–RNA complexes (pR) formed in 0, 25, or 50 mM NaCl. Untreated CD1c^+^ DCs or IL-3 treated pDCs were used as negative controls, while R848 was used as a control for TLR7/8 stimulation and poly I:C and CpG-C were used as positive controls for CD1c^+^ DCs or pDCs, respectively. **a** The cell viability was determined by flow cytometry. The mean percentage ± SEM of cells negative for live–dead marker from 7–8 CD1c^+^ DC donors and 6–8 pDC donors is depicted. **b**, **c** The relative expression of MHC class I and HLA-DR (**b**) and CD86 **(c)** on viable cells was calculated by normalizing the MFI values for each donor against the negative control. The fold increase ± SEM of 6–10 CD1c^+^ DC and pDC donors is depicted. Wilcoxon matched-pair signed-rank tests were performed on raw data, comparing against negative control, and are indicated by *(*p* < 0.05), **(*p* < 0.01), ***(*p* < 0.001), or NS (non-significant)

To evaluate the ability of RNA complexed to protamine to activate DCs, we formulated protamine–RNA complexes with different salt conditions (Fig. 2). Purified DCs were cultured overnight with concentrations ranging from 1.5 to 15 µg/ml of protamine–RNA complexes formed in either 0, 25, or 50 mM NaCl. As a control for cell stimulation, the TLR7/8 ligand R848 was used. Viability and expression of maturation markers were investigated. Unstimulated pDCs do not survive ex vivo; therefore, IL-3-treated cells were used as a negative control [28].

The viability of the CD1c^+^ DCs was not affected by protamine–RNA complexes, while a slight decrease in viability was detected for pDCs (Fig. 2a). To investigate whether protamine–RNA complexes had a direct toxic effect on the pDCs, IL-3 was added to the cultures and the viability examined. There was no difference in viability between R848-treated pDCs and protamine–RNA-treated pDCs. IL-3 had a favorable effect on pDC viability in the tested conditions (Supplementary Fig. 1a).

Next, the ability of the protamine–RNA complexes to mature DCs was investigated. For the CD1c DCs, all complexes increased the expression of MHC class I, while only smaller complexes had this effect on pDCs (Fig. 2b). Protamine–RNA-induced upregulation of HLA-DR was detected on the CD1c^+^ DCs, while on the pDCs, IL-3 alone increased HLA-DR expression and no additive effect of the complexes was observed. All complexes induced upregulation of maturation marker CD86 on CD1c^+^ DCs, with the strongest effect detected for the large complexes formed in the presence of high salt concentrations (Fig. 2c). For the pDCs, an opposite pattern was seen; the highest upregulation of CD86 was induced by protamine–RNA complexes formed without salt (Fig. 2c).

Since the viability of the DCs did not differ between the protamine–RNA concentrations used and a dose-dependent upregulation of maturation markers and MHC complexes was observed, we performed additional experiments with the highest concentration tested. This concentration also led to upregulation of additional activation markers, such as CD40, CD80, and CCR7 (Supplementary Fig. 1b, c). Unprotected RNA has previously been shown to activate an antigen-specific immune response upon intranodal injection [29]. However, addition of RNA or protamine without complexing did not lead to DC maturation in our hands (data not shown).

### Dendritic cells stimulated with protamine–RNA complexes release high levels of pro-inflammatory cytokines

**Fig. 3:**
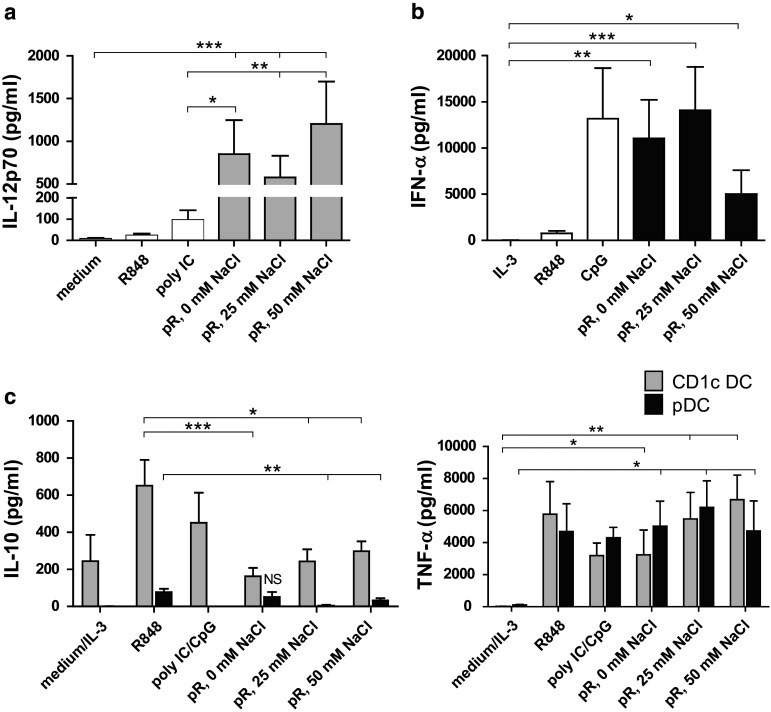
Pro-inflammatory cytokines are secreted by protamine–RNA-stimulated DCs. The concentrations of pro-inflammatory cytokines in supernatants taken from CD1c^+^ DCs and pDCs stimulated overnight with medium or IL-3, R848, poly I:C or CpG-C, or 15 µg/ml protamine–RNA complexes (pR) formed in 0, 25, or 50 mM NaCl were measured by ELISA. The concentration ± SEM of IL-12p70 (**a**) from 9 to 11 CD1c^+^ DC donors, IFN-α (**b**) from 6–10 pDC donors, or IL-10 and TNF-α (**c**) from 5-11 CD1c^+^ DC and 4–8 pDC donors is depicted. Wilcoxon matched-pair signed-rank tests were performed between indicated groups and are indicated by *(*p* < 0.05), **(*p* < 0.01), or ***(*p* < 0.001)

The cytokine profile of activated DCs plays an important role in the skewing of naïve T cells. We therefore compared the release of pro-inflammatory cytokines from purified DCs stimulated overnight with protamine–RNA complexes formed in water or increasing salt concentrations. All three protamine–RNA complex formations induced fivefold to tenfold more IL-12p70 than the positive control poly I:C (Fig. 3a). pDCs secreted comparable levels of IFN-α upon stimulation with the positive control CpG-C and protamine–RNA complexes (Fig. 3b). In accordance with the results in Fig. 2, the smaller protamine–RNA complexes induced the strongest response in pDCs.

An important regulator of IL-12 production is IL-10 [30]. IL-10 production by both CD1c^+^ DCs and pDCs was observed after stimulation with poly I:C and CpG-C, respectively. Significantly lower amounts of IL-10 were observed in the protamine–RNA-stimulated cultures (Fig. 3c). Both DC subsets secreted significant amounts of TNF-α upon protamine–RNA treatment (Fig. 3c).

### Protamine–RNA complexes induce TLR signaling via the endosomal compartment of DCs

**Fig. 4:**
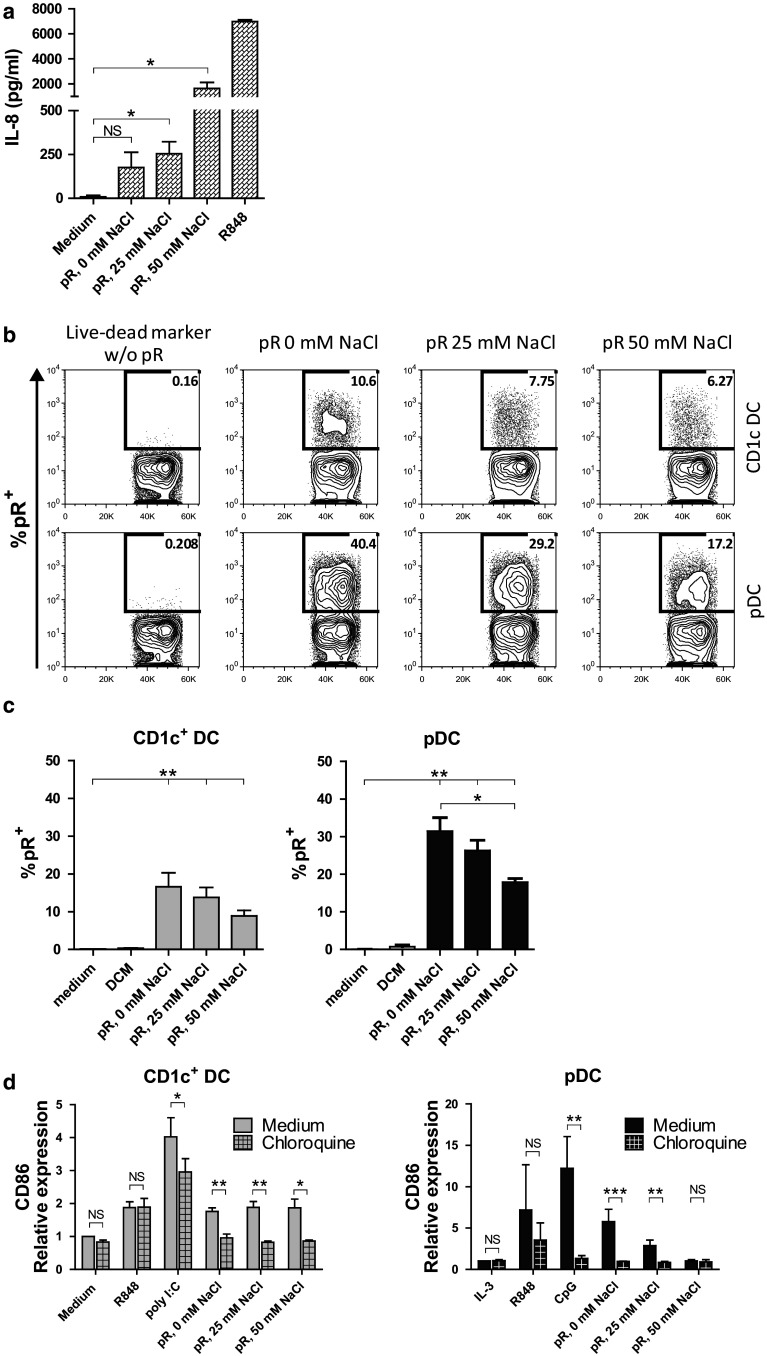
Effect of protamine–RNA complexes is TLR mediated. **a** TLR8 expressing HEK cells were cultured overnight with medium alone, R848, or protamine–RNA complexes (pR) formed in 0, 25, or 50 mM NaCl. The mean results ± SEM from three individual experiments run in triplicate or duplicate is depicted. *T* tests were performed on pooled data between indicated groups and are indicated by *(*p* < 0.05) or NS (non-significant). (**b-c**) DCs were cultured for 1 h with live–dead marker-labeled protamine–RNA complexes (pR) formed in 0, 25, or 50 mM NaCl or with live–dead marker alone and analyzed by flow cytometry. Percentages of protamine–RNA-positive cells were calculated on gated DCs and depicted as a representative figure (**b**) or as the mean uptake ± SEM from 7 CD1c^+^ DC and pDC donors (**c**). Wilcoxon matched-pair signed-rank tests were performed between indicated groups and are indicated by *(*p* < 0.05) or **(*p* < 0.01). **d** CD1c^+^ DCs and pDCs were pre-incubated for 1 h with chloroquine before the addition of medium alone or IL-3, R848, poly I:C or CpG-C, or protamine–RNA complexes (pR) formed in 0, 25, or 50 mM NaCl. The upregulation of CD86 was measured by flow cytometry after overnight culture and the relative expression was calculated by normalizing the MFI values for each donor against the negative control. Fold increase ± SEM of 4–5 CD1c^+^ DC and 3–5 pDC donors is depicted. *T* tests were performed on raw data between indicated groups and are indicated by *(*p* < 0.05), **(*p* < 0.01), ***(*p* < 0.001), or NS (non-significant)

When investigating the cytokine profile of protamine–RNA-stimulated DCs, a shift toward NFκB-driven responses was seen with increasing salt concentrations, while a type I IFN response was seen when the complexes were formed in water. We therefore investigated what mechanism was driving the DC responses. The activation via TLR7 in response to protamine–RNA complexes is previously shown in a murine setting [20],

We confirmed that the complexes had the ability to signal via TLRs by testing the effect of the complexes on TLR8-expressing HEK cells. TLR8-expressing or non-transduced HEK cells were cultured overnight with the different protamine–RNA complexes, and IL-8 was measured in the supernatant. While no IL-8 was secreted from TLR8 non-expressing cells (data not shown), all protamine–RNA complexes were able to induce IL-8 production in TLR8-expressing HEK cells, with the complexes formed with high salt concentrations inducing the highest response (Fig. 4a).

To activate TLR7/8, the ligand should reach the endosomes, where signaling is initiated by lowering the pH. To study the binding and uptake of protamine–RNA complexes by DCs, protamine–RNA complexes were incubated with a fixable live–dead dye. This dye binds nucleic acids, and its activity can be stopped by addition of a protein blocker. Protamine–RNA complexes labeled with the live–dead dye, the live–dead dye alone, or medium alone were added to CD1c^+^ DCs and pDCs. After culturing for 1 h, the binding and uptake was assessed by flow cytometry (Fig. 4b, c). In some experiments, propidium iodide was used as a viability stain to confirm that the labeled complexes were bound to viable DCs (data not shown). Approximately 15 % of the CD1c^+^ DCs associated with labeled complexes after 1 h of culture. For pDCs, the smallest complexes were most efficiently bound, although also the larger complexes formed in high NaCl concentrations associated stronger with pDCs as compared to CD1c^+^ DCs.

Next, we investigated the importance of endosomal maturation for protamine–RNA-induced DC activation. Endosomal acidification was prevented using chloroquine 1 h before addition of the complexes. The TLR ligands poly I:C and CpG-C were used as controls for the efficacy of chloroquine treatment, while R848, previously shown to be able to induce DC activation despite chloroquine treatment [31], was used as a positive control. The increase in CD86 and CD80 expression was inhibited when the chloroquine pre-treated DCs were cultured with the complexes, as compared to untreated cells (Fig. 4d and Supplementary Fig. 2a). The secretion of IL-12p70 and IFN-α was absent in chloroquine-treated CD1c^+^ DCs and pDCs, respectively, as well as TNF-α (Supplementary Fig. 2b). Although the viability of the CD1c^+^ DCs was not strongly affected by the addition of chloroquine, pDCs were deprived of their survival signal and a decreased viability was seen in all groups except for the IL-3- or R848-treated cells (Supplementary Fig. 2c), further demonstrating the importance of the endosomal route in response to protamine–RNA complexes.

### Protamine–RNA-stimulated DCs induce proliferation and activation of T cells

**Fig. 5:**
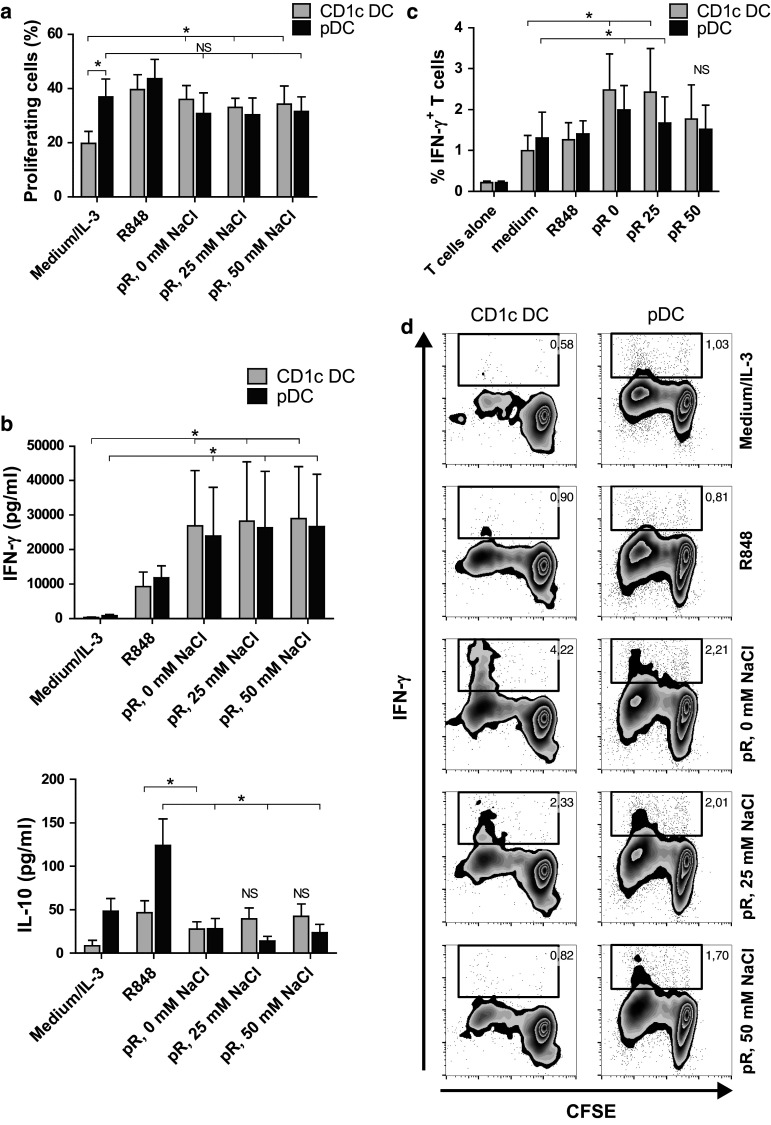
T cell proliferation and IFN-γ production is induced by protamine–RNA-stimulated DCs. DCs were cultured with medium alone or IL-3, R848, or protamine–RNA complexes (pR) formed in 0, 25, or 50 mM NaCl. CFSE-labeled allogenic PBLs or T cells were added after overnight culture. **a** The dilution of CFSE-labeled PBLs was measured on CD3^+^CD11c^−^BDCA2^−^ cells by flow cytometry on day 5. Cultured, unstimulated PBLs were used as reference. The mean percentage proliferating cells ± SEM of 6 CD1c^+^ DC and pDC donors is depicted. **b** The expression of IFN-γ and IL-10 in day 2 DC:PBL co-cultures was measured with ELISA. Mean concentration ± SEM from 5 to 6 CD1c^+^ DC and pDC donors is depicted. **c**, **d** Intracellular IFN-γ was measured in CD3^+^CD11c^−^BDCA2^−^ T cells cultured for 5 days either alone or with DCs stimulated with medium or IL-3, R848, or pR complexes. Results are depicted as the mean percentage IFN-γ^+^ cells ± SEM from 6 CD1c^+^ DC and pDC donors (**c**) or as a representative figure (**d**). Wilcoxon matched-pair signed-rank tests were performed between indicated groups and are indicated by *(*p* < 0.05), **(*p* < 0.05), or NS (non-significant)

After characterizing the effects of protamine–RNA on primary DCs, the subsequent T cell response induced by activated DCs was investigated. Proliferation of CFSE-labeled PBLs was measured in an allogeneic setting. The percentage of dividing CD3^+^ cells was determined 5 days after addition of protamine–RNA-activated DCs. As a positive control for proliferation, SEB was added (data not shown). R848- and protamine–RNA-treated CD1c^+^ DCs induced comparable proliferation of T cells, which was significantly higher than untreated DCs (Fig. 5a). Stimulated pDCs also induced a proliferative response in T cells comparable to IL-3-treated cells (Fig. 5a).

To investigate the response of the stimulated T cells, the release of cytokines was measured. Supernatants were taken at day 2 of DC:PBL co-cultures, and IFN-γ, IL-10, and IL-5 were measured. Protamine–RNA-stimulated pDCs induced more IFN-γ than IL-3-treated cells and in similar quantities as protamine–RNA-stimulated CD1c^+^ DCs (Fig. 5b). In addition, the PBLs produced less IL-10 when co-cultured with protamine–RNA-stimulated pDCs than with IL-3 treated cells. For CD1c^+^ DCs, the negative control induced only low levels of IFN-γ and IL-10, while protamine–RNA-treated DCs stimulated elevated levels of both cytokines (Fig. 5b). No IL-5 secretion was detected (data not shown). This supports that T cells activated by protamine–RNA-stimulated DCs display Th1 characteristics and not a regulatory profile. To confirm this, the MLR experiment was repeated with purified allogeneic T cells, which were investigated for intracellular IFN-γ production (Fig. 5c, d). T cells co-cultured with DCs activated by the smaller complexes produced significantly more IFN-γ than control cultures.

### Antigen-specific T cell responses are induced by protamine–RNA-stimulated DCs

**Fig. 6:**
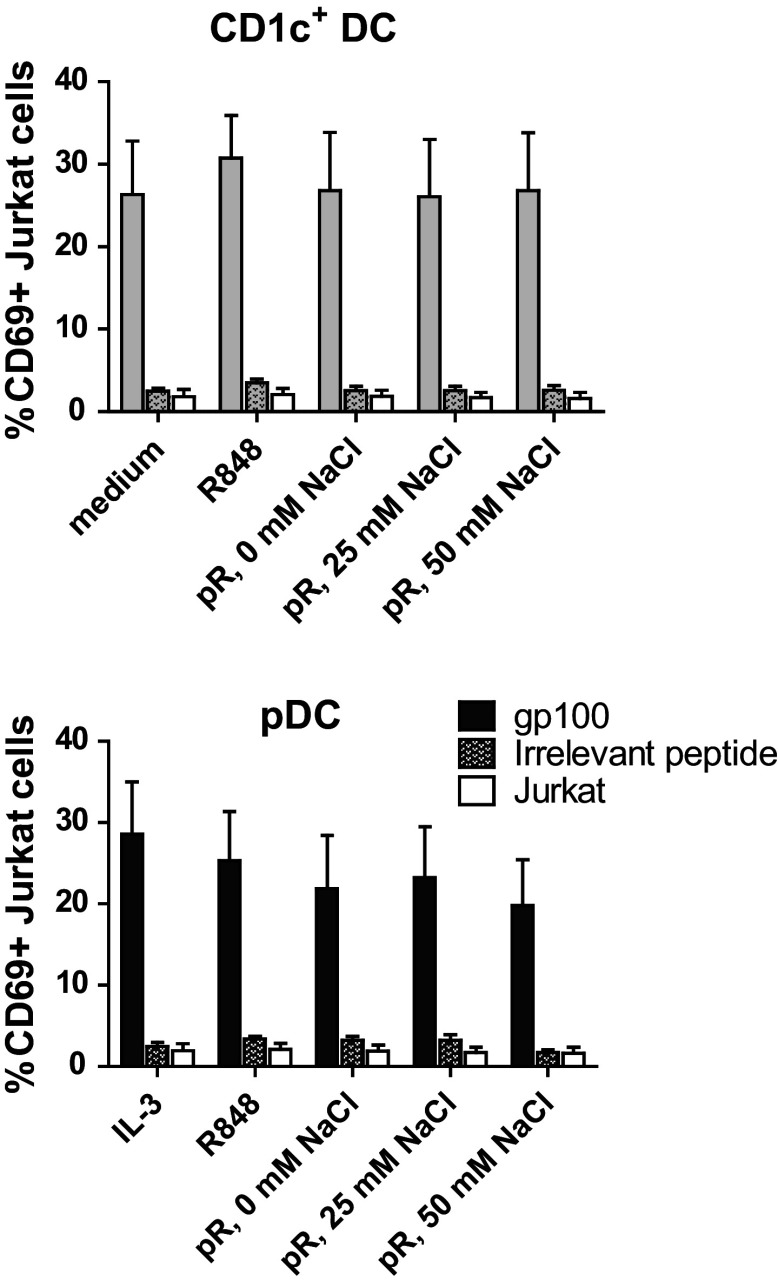
Protamine–RNA-stimulated DCs can induce antigen-specific T cell responses. HLA-A0201^+^ DCs were pulsed with gp100_280–288_ short peptide or irrelevant peptide, stimulated with protamine–RNA complexes (pR) formed in 0, 25, or 50 mM NaCl, and co-cultured overnight with Jurkat cells expressing the TCR-v-beta14 receptor specific for the gp100_280–288_ peptide in the context of HLA-A0201. The upregulation of CD69 on Jurkat cells was assayed with flow cytometry. The mean percentage of CD69 expression ± SEM on Jurkat cells co-cultured with 6 CD1c^+^ DC and pDC donors is depicted

To test the ability of protamine–RNA-stimulated DC to present antigens to T cells, HLA-A0201^+^ DCs were pulsed with either gp100_280–288_ short peptide or irrelevant peptide and subsequently cultured with Jurkat cells transfected with the TCR-v-beta14 receptor recognizing gp100_280–288_ in the context of HLA-A0201 (Fig. 6). The Jurkat cells are activated upon recognition of the specific antigen, independent of co-stimulatory signals. A distinct upregulation of activation marker CD69 was detected in Jurkat cells cultured with gp100_280-288_-pulsed DCs, while irrelevant peptide-pulsed DCs induced responses comparable to Jurkat cells cultured with the protamine–RNA complexes in the absence of DCs. Hence, protamine–RNA-treated pDCs and CD1c^+^ DCs both retain the ability to present specific antigens to T cells.

## Discussion

Our research group has recently focused on developing immunotherapeutic strategies based on primary DC subsets, using different clinical activation protocols for mDCs and pDCs [8, 9]. In the pDC trial, Frühsommer-meningoencephalitis (FSME) vaccine was used to activate the cells [9, 32], while unbeneficial responses were seen for CD1c^+^ DCs treated with FSME. Instead, GM-CSF has been used to activate this DC population, which does not efficiently activate pDCs to produce type I IFNs (Schreibelt et al., manuscript in preparation) [8]. The activation status of DCs is an important factor for the subsequent adaptive immune responses. To induce full activation of DCs, signaling via PRRs such as TLRs is necessary [3, 33]. Although a vast number of TLR ligands have been developed, only a few are approved for clinical use. Currently, there is no TLR ligand available that can be used to activate both mDCs and pDCs which is produced according to good manufactoring practice (GMP) [18]. Therefore, we have used the clinical grade reagents protamine and mRNA to compile a TLR7/8 ligand with the ability to activate both pDCs and CD1c^+^ DCs.

The immunopotent effect of ssRNA complexed to cationic proteins has previously been described [19, 20, 34], and the ionic content of the solution in which the complexes are formed has shown to affect their features and size [26]. Here, we have investigated the stimulatory effect of protamine–RNA complexes formed either in water or in increasing salt concentrations. Water or low salt concentrations formed smaller complexes, while high salt concentrations mediated formation of complexes larger than 500 nm. When adding these complexes to primary pDCs and CD1c^+^ DCs, the two subsets responded differently. All three complex formulations could stimulate CD1c^+^ DCs to upregulate maturation markers, MHC complexes, and pro-inflammatory cytokine production, although highest responses were seen with the larger complexes. Oppositely, pDCs fully matured after addition of the smaller complexes and not consistently with the complexes formed in high salt concentrations. This might be due to the inability of pDCs to efficiently engulf larger particles [27]. Indeed, when comparing the ability to bind and take up protamine–RNA complexes, pDCs associated significantly better with smaller complexes than with the large ones, while CD1c^+^ DCs associated with the different complexes equally. However, pDCs did associate with the large complexes, indicating that the binding and uptake is not the only factor regulating the cellular responses toward the complexes. For instance, the larger protamine–RNA complexes might be stronger ligands for TLR8, while smaller complexes are more efficient for TLR7 stimulation. This phenomenon has previously been described for synthetic TLR7/8 ligands [35, 36]. Furthermore, a difference in proteolytic cleavage between TLR7 and TLR8 was recently demonstrated [37]. In addition, the observed shift from a type I IFN-mediated response toward a NFκB-driven response has been reported to differentiate the responses mediated by TLR7 and TLR8 activation [36]. Although positive responses were detected for all three types of complexes, the TLR8 expressing HEK cell line responded strongest to the largest ones. Nevertheless, despite weaker responses, pDCs treated with protamine–RNA complexes formed in high salt concentration were still viable without extra addition of IL-3, which is needed for the long-term culturing of non-stimulated pDCs [28]. This indicates that even the larger complexes have the ability to provide a survival signal, but not necessarily fully activate pDCs. The decrease in pDC viability upon inhibition of endosomal acidification further supports this hypothesis.

To act as an effective adjuvant, a functional activation of relevant adaptive responses must be induced by DC treated with the stimulus. Protamine–RNA-stimulated DCs indeed induced proliferation of T cells. For CD1c^+^ DCs, a significant difference was observed between unstimulated and stimulated DCs. Addition of the survival factor IL-3 to pDCs resulted in a high proliferation of PBLs, most likely due to an IL-3-induced upregulation of antigen-presenting molecules. We therefore investigated the activation of the co-cultured cells to determine what kind of adaptive response protamine–RNA-treated DCs induced. An increase in IFN-γ production could be detected in both CD1c^+^ DC and pDC co-cultures, where T cells stimulated with protamine–RNA-treated DCs produced the highest levels of the cytokine.

In addition to inducing IFN-γ-producing T cells, an antigen-specific immune response must also be induced by the activated DCs. In our vaccination setting, stimulated DCs are pulsed externally with peptide antigens [8, 9, 38]. Protamine–RNA-treated DCs induced an antigen-specific response when pulsed with the gp100_280–288_ HLA-A0201-binding peptide. IL-3-treated pDCs gave stronger results than both protamine–RNA complexes and R848 treatment, probably due to the elevated expression of antigen-presenting molecules, and it should be noted that this assay is not dependent on the activation status of the APC but rather on their ability to present peptide antigens.

To conclude, protamine–RNA complexes have the ability to induce maturation of pDCs and CD1c^+^ DCs via endosomal-dependent pathways, most likely via ssRNA-mediated activation of TLR7 and TLR8. For CD1c^+^ DCs, the highest responses were observed with complexes of larger size, but significant upregulation of maturation markers, MHC molecules, and pro-inflammatory cytokines was detected also for the smaller complexes. Importantly, protamine–RNA complexes induced release of the Th1-skewing cytokine IL-12p70 from CD1c^+^ DCs, making the stimulus highly interesting when an anti-cancer response is desired. Also pDCs responded to protamine–RNA complexes, but for this subset smaller complexes, formed in water or low salt concentrations, induced the strongest maturation as well as IFN-α production. Taken together, protamine–RNA complexes pose as an interesting adjuvant that can be GMP produced and used for activation of primary DCs in cell-based immunotherapy. Depending on the protocol, large complexes can be used to activate CD1c^+^ DCs, while small complexes are suitable to stimulate pDCs. Since the complexes are able to activate both CD1c^+^ DCs and pDCs, it also opens for the possibility of using protamine–RNA as a stimulus in a vaccine consisting of the two DC subsets together. Then the smaller complexes, with the ability to activate both pDCs and CD1c^+^ DCs, are recommended. High levels of IFN-α have been shown to activate cytotoxic responses in natural killer cells [39, 40], but have also been implemented in pDC:mDC cross-talk and Th1 responses [15, 41, 42]. The most prominent Th1-skewing cytokine, IL-12p70, is secreted by protamine–RNA-treated CD1c^+^ DCs. The combination of IFN-α and IL-12p70 derived from protamine–RNA-stimulated pDCs and mDCs is a potent and multifaceted immunotherapy.

### Electronic supplementary material

Below is the link to the electronic supplementary material.

Supplementary material 1 (PDF 345 kb)

## Abbreviations

CCR7: C-C chemokine receptor type 7
CFSE: Carboxyfluorescein diacetate succinimidyl ester
CTL: Cytotoxic CD8^+^ T cell
DC: Dendritic cell
FSME: Frühsommer-meningoencephalitis
GMP: Good manufactoring practice
HLA: Human leukocyte antigen
IFN: Interferon
IL: Interleukin
MFI: Mean fluorescence intensity
MHC: Major histocompatibility complex
MLR: Mixed lymphocyte reaction
mAb: Monoclonal antibody
mDC: Myeloid DC
PBL: Peripheral blood leukocyte
PBMC: Peripheral blood mononuclear cell
pDC: Plasmacytoid DC
poly I:C: Polyinosine-polycytidylic acid
pR: Protamine–RNA complex
PRR: Pattern recognition receptor
SEB: Staphylococcal enterotoxin B
ssRNA: Single-stranded RNA
TCR: T cell receptor
Th: T helper
TLR: Toll-like receptor
TNF: Tumor necrosis factor
