# Supplementary material for: Protamine-stabilized RNA as an ex vivo stimulant of primary human dendritic cell subsets
Source: Cancer Immunol Immunother. 2015 Aug 15;64(11):1461–73. doi: 10.1007/s00262-015-1746-9 (PMC4612318; doi:10.1007/s00262-015-1746-9)
Supplement: Supplementary file 1 — Supplementary material 1 (PDF 345 kb) [file 262_2015_1746_MOESM1_ESM.pdf]

Supplementary figure 1

a

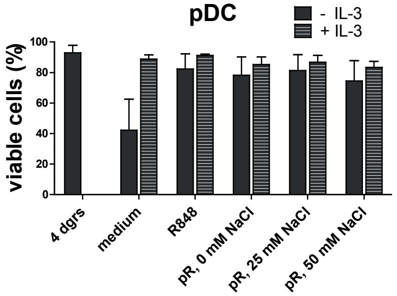

b

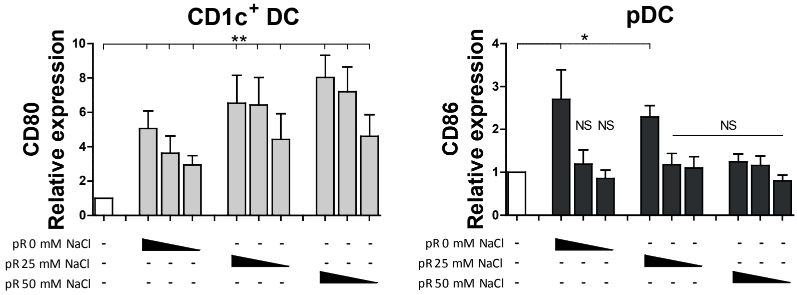

c

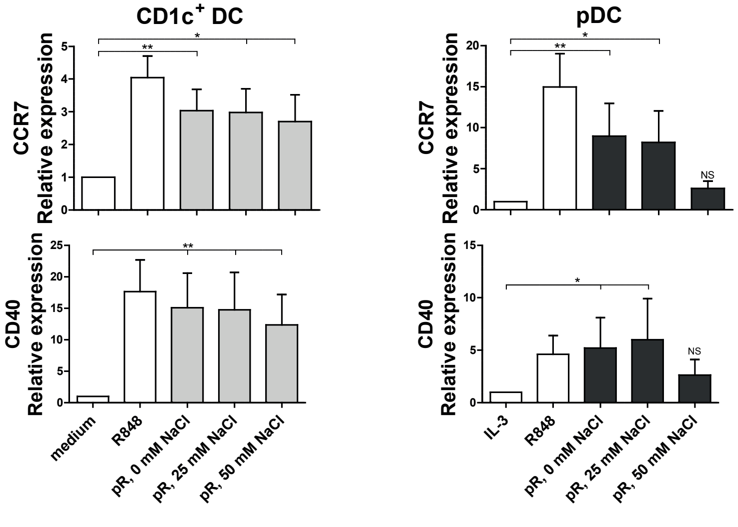

Supplementary figure 1: pDC viability in the presence or absence of IL-3 and DC maturation upon stimulation with protamine:RNA complexes

Purified CD1c+ DCs and pDCs were cultured 18-24h with 15 µg/ml, 7.5 µg/ml or 1.5 µg/ml of protamine:RNA complexes (pR) formed in 0, 25, or 50 mM NaCl.(**a**) The viability of pDCs cultured with R848 or 15 µg/ml of pR in the presence or absence of IL-3 was determined. The mean percentage ± SEM of cells negative for live-dead marker from 4 pDC donors is depicted. (**b**) The relative expression of CD80 on DCs treated with medium or IL-3 or increasing concentration of pR is calculated by normalizing the MFI values for each donor against the negative control. The fold increase ± SEM of 9-10 CD1c+ DC donors and 4-5 pDC donors is depicted. (**c**) The relative expression of CD40 and CCR7 from 7 CD1c+ DC and pDC donors treated with medium or IL-3, R848, or 15 µg/ml of pR is depicted. Wilcoxon matched-pair signed rank tests were performed on raw data, comparing against negative control, and are indicated by * ($p<0.05$), ** ($p<0.01$), or NS (non-significant)

Supplementary figure 2

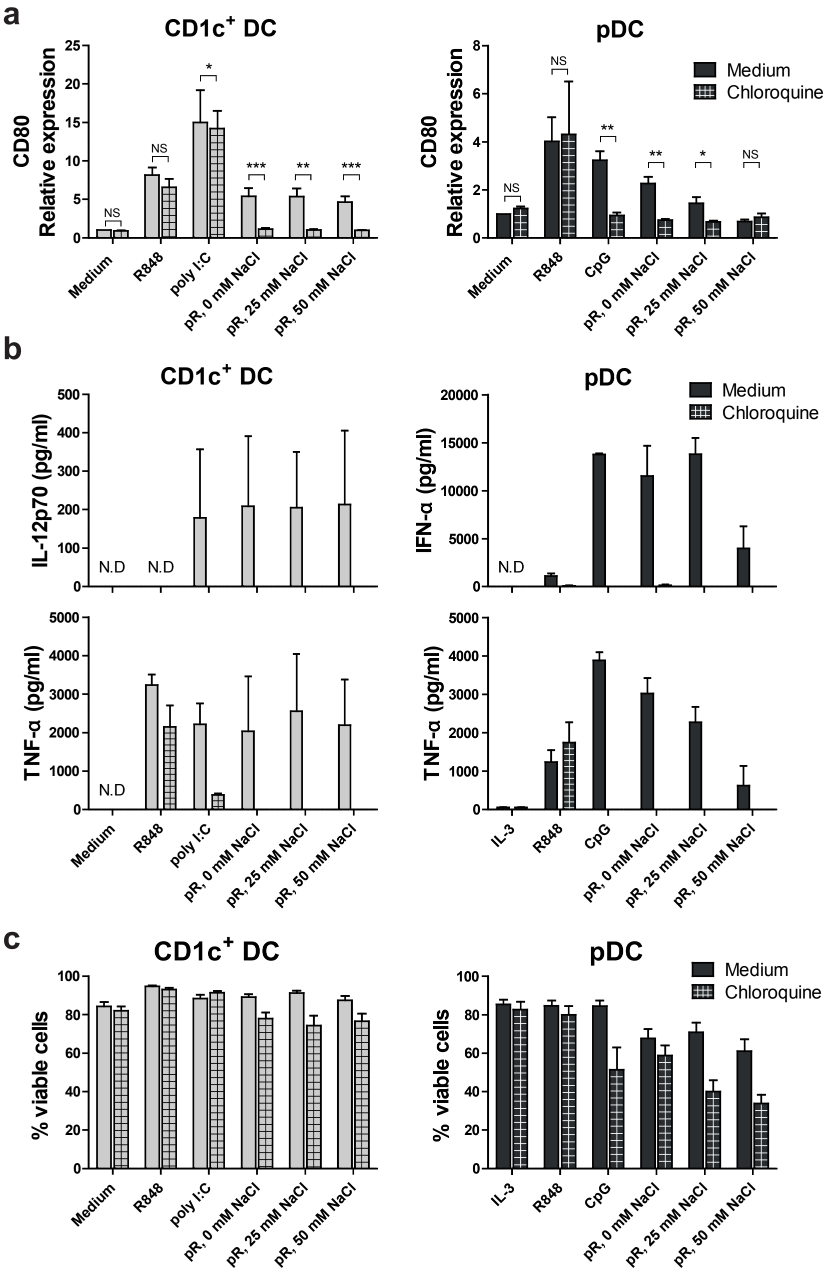

Supplementary figure 2: Activation and viability of chloroquine-treated DCs

CD1c$^+$ DCs and pDCs were pre-incubated for 60 minutes with chloroquine before the addition of medium or IL-3, R848, poly I:C or CpG-C, or protamine:RNA complexes (pR) formed in 0, 25, or 50 mM NaCl. (**a**) The relative expression of CD80 on DCs was calculated by normalizing the MFI values for each donor against the negative control. Fold increase ± SEM of 4-5 CD1c$^+$ DC and pDC donors is depicted. Wilcoxon matched-pair signed rank tests were performed and are indicated by * ($p<0.05$), ** ($p<0.01$), *** ($p<0.001$), or NS (non-significant). (**b**) The secretion of IL-12p70 from 3 CD1c$^+$ DC donors, of IFN-α from 2-3 pDC donors, and of TNF-α from the same donors is depicted as mean percentage ± SEM. N.D=not detected. (**c**) The viability of DCs cultured with chloroquine is analyzed and the mean percentage ± SEM of viable cells from 5 CD1c$^+$ DC donors and 4 pDC donors is depicted.
